# Development of a Three-Dimensional Mucosal Surface Cast of the Caprine *Ruminoreticulum*

**DOI:** 10.3390/vetsci13040390

**Published:** 2026-04-17

**Authors:** Joachim Truelsen, Julia Hollenbach, Elisabeth Engelke, Matthias Lüpke, Kerstin von Pückler, Lara Ott, Johanna-Marie Haumann, Sandra Wissing, Kristin Elfers, Christiane Pfarrer

**Affiliations:** 1 Institute for Anatomy, University of Veterinary Medicine Hannover, Bischofsholer Damm 15, 30173 Hannover, Germany; joachim.truelsen@tiho-hannover.de (J.T.); elisabeth.engelke@tiho-hannover.de (E.E.); christiane.pfarrer@tiho-hannover.de (C.P.); 2 Institute of General Radiology and Medical Physics, University of Veterinary Medicine Hannover, Bischofsholer Damm 15, 30173 Hannover, Germany; matthias.luepke@tiho-hannover.de; 3 Clinic for Small Animals, University of Veterinary Medicine Hannover, Bünteweg 9, 30559 Hannover, Germany; kerstin.von.pueckler@tiho-hannover.de; 4 Clinical Skills Lab, University of Veterinary Medicine Hannover, Bischofsholer Damm 15, 30173 Hannover, Germany; lara.ott@tiho-hannover.de (L.O.); johanna-marie.haumann@tiho-hannover.de (J.-M.H.); sandra.wissing@tiho-hannover.de (S.W.); 5 Institute of Physiology and Cell Biology, University of Veterinary Medicine Hannover, Bischofsholer Damm 15, 30173 Hannover, Germany; kristin.elfers@tiho-hannover.de

**Keywords:** 3D model, 3D printing, mucosal surface cast, *Ruminoreticulum*, veterinary anatomy

## Abstract

In veterinary education, many exercises are performed on animals. Palpating the mucosa of the *Ruminoreticulum* in ruminants is a necessary preparatory exercise for future surgery. However, there are legal and ethical obligations to reduce the use of animals and improve animal welfare. This can be achieved using 3D models and simulators. To allow students to practice palpating the goat’s forestomach, a simulator is being developed. The aim of the present study was to produce replicas of the mucosal surface of the *Ruminoreticulum* for the inner lining of this simulator. Two methods were applied and compared: 3D printing and surface casting. For 3D printing, computed tomography-based virtual templates were created and printed after appropriate post-processing. For the surface cast, a negative mold of the mucosal surfaces was created using epoxy resin. The positive mucosal cast was then created using silicone. The results showed a clear advantage of surface casting compared to 3D printing. The virtual templates and 3D prints lacked fine anatomical structures. In contrast, the surface casting method yielded detailed replicas of the mucosal surfaces of *Rumen* and *Reticulum*, including even finer anatomical structures. Since the silicone casts also allowed for haptic differentiation of mucosal formations, they can be considered a suitable inner lining for the planned simulator.

## Simple Summary

Practical training on animals is essential for veterinary education. However, there are legal and ethical obligations to reduce the use of animals and improve the welfare of those used. To achieve this, 3D models and simulators can be used to allow students to learn certain techniques. In that way, a 3D simulator of a goat is being developed to allow students to practice palpating the two forestomach compartments, *Rumen* and *Reticulum*, before performing this procedure on living rumen-fistulated ruminants. For blind tactile orientation, it is important to replicate the inner mucosal surface of the *Ruminoreticulum* as lifelike as possible. To this end, two methods were tested and compared: 3D printing and mucosal surface casting. For 3D printing, computed tomography-based virtual templates of the mucosal surfaces were generated and printed. For surface casting, epoxy resin-based negative molds of the mucosal surfaces were created and positive casts were produced using self-hardening silicone. The technique of mucosal surface casting showed clear advantages over 3D printing. The virtual templates and 3D prints lacked anatomical details. In contrast, silicone casts of the mucosal surface accurately reproduce the anatomical structures of the organs and allow for tactile differentiation which makes them suitable for the planned simulator.

## 1. Introduction

Achieving precise anatomical and physiological knowledge is fundamental in veterinary education. To this end, the use of animals and cadavers has been an integral part of the veterinary medicine curriculum for centuries [1]. In the preclinical part of veterinary education, for example, palpation of living, rumen-fistulated ruminants is an integral part of physiology courses and is also an essential preparation for future surgical procedures involving the *Rumen*. However, the goal of intensive and thorough training conflicts with the legal and ethical mandate to reduce the use of animals in training and improve animal welfare [2,3]. To meet this demand, the use of 3D models and simulators in medical education has become increasingly important [4,5,6]. Their use leads to a reduction in the number of animals and enables students to learn certain techniques before performing them on live animals.

3D printing has proven its potential as an animal-reduced resource for veterinary anatomical education in different applications [1,7,8,9]. Access to this technology is now easy due to affordable 3D printers and open-source Computer-Aided Design (CAD) programs [10] for creating virtual templates based on surface scans [8] and medical imaging [9]. While hard tissue specimens like bones can be manufactured with high accuracy [7], the production of soft tissue specimens remains a frontier, primarily due to poor depiction by imaging techniques which form the basis for any image and, in turn, for the resulting 3D print [11]. Moreover, imitation of the soft tissue’s biomechanical properties is challenging [6].

Another 3D modeling approach is corrosion cast preparation. With this technique, hollow anatomical structures are filled with self-hardening fluids to create a negative replica. Afterwards, the surrounding organ tissue is removed by preparation and/or maceration to obtain the cast as a model [12]. Corrosion casting is commonly used to create replicas of cardiovascular, biliary, tracheo–bronchial or urogenital systems [13,14,15,16]. However, the creation of surface casts of anatomical structures is also possible. This works by first taking a negative mold of the anatomical structure, which is then filled with the casting material in a second step. The result is an exact copy of the original anatomical structure [12].

The aim of the current study was to produce a replica of the mucosal surface of the two forestomach compartments *Rumen* and *Reticulum* of a goat as detailed and lifelike as possible. This replica will later be used as the inner lining in a plastic 3D model of the forestomach of a goat, which is designed to allow students to practice palpating the *Rumen* and *Reticulum* before working on living animals. To achieve this goal, two methods were compared: (1) the creation of virtual templates followed by 3D printing, and (2) the generation of surface casts.

## 2. Materials and Methods

### 2.1. Specimens

The *Ruminoreticulum* of one fully grown goat (German Improved Fawn, female) was used for creating both virtual templates and negative molds of forestomach subsections. The goat was euthanized for research purposes in accordance with section 4 (3) of the German Animal Protection Law (approval number: TiHo T-2025-10). The extraction of the organ and the removal of the ingesta were performed shortly after euthanasia.

### 2.2. Creation of the Virtual Template and 3D Printing

#### 2.2.1. Medical Imaging

Computed tomography (CT) scans were used to generate virtual templates within 5 h postmortem. The goat’s *Rumen* and *Reticulum* were separated from each other, cut open, and were laid out flat with the mucosa facing upwards. The scans were acquired using a spectral detector CT (Philips IQon Spectral CT, Philips Healthcare, Hamburg, Germany). The used settings were: 0.7 mm slice thickness, 120 kV peak, and an X-ray tube current of 120 mA for 100 s resulting in 0.7 mm^3^ voxel size. Generating 3D prints from medical imaging data requires three steps: image segmentation, mesh refinement and 3D printing [17].

#### 2.2.2. Image Segmentation

The CAD program Amira-Avizo^®^ (version 2023.2, Life Technologies GmbH, Darmstadt, Germany) was used for image segmentation. A semi-automatic thresholding approach with manual delineation was used to separate the organ pieces from the patient table to sufficiently segment the region of interest. A broad thresholding range was chosen to include as many details of the mucosal surface as possible. Manual delineation in some slices was necessary because the range of the threshold included the patient table.

#### 2.2.3. Mesh Refinement

The resulting segmentation was expressed as a superficial mesh by the command “generate surface” using Amira-Avizo^®^. By conversion, a moderate global smoothing filter was applied for mesh refinement. This was necessary to reduce the staircasing errors in the virtual 3D model caused by the resolution of the original images [17]. To further process the virtual templates, they were exported as stereolithography (STL) data.

#### 2.2.4. 3D Printing

The STL files were imported into the slicing software PrusaSlicer (Version 2.9.4, Prusa Research a.s., Prague, Czech Republic) to prepare the virtual model for 3D printing. Before printing, the lengths of the virtual models` three-dimensional axes were reduced by 50% to create a 1:2 sized test print. The 3D model was printed using the HT500 FDM printer (Kühling&Kühling GmbH, Trappenkamp, Germany) with a layer thickness of 0.1 mm and 20% infill. The material used for printing was Extrudr^®^ (PLA NX2, Lauterach, Austria).

### 2.3. Creation of the Mucosal Surface Cast

A total of eight organ pieces of the *Ruminoreticulum* served as templates for the surface cast.

#### 2.3.1. Negative Mold

For the creation of the negative mucosal mold the epoxy resin BIODUR^®^ E20 PLUS, RED (BIODUR Products GmbH, Heidelberg, Germany) was used due to its low viscosity, resistance to alkalis and its slight elasticity after curing [12,14,15]. Methylethylketone (MEK) was used to further reduce the viscosity while maintaining the mold material’s original features.

The mixture used consisted of 1 kg BIODUR^®^ E20 PLUS, 0.55 kg BIODUR^®^ E20 PLUS HARDENER and 100 g MEK as recommended by the manufacturer.

**Figure 1:**
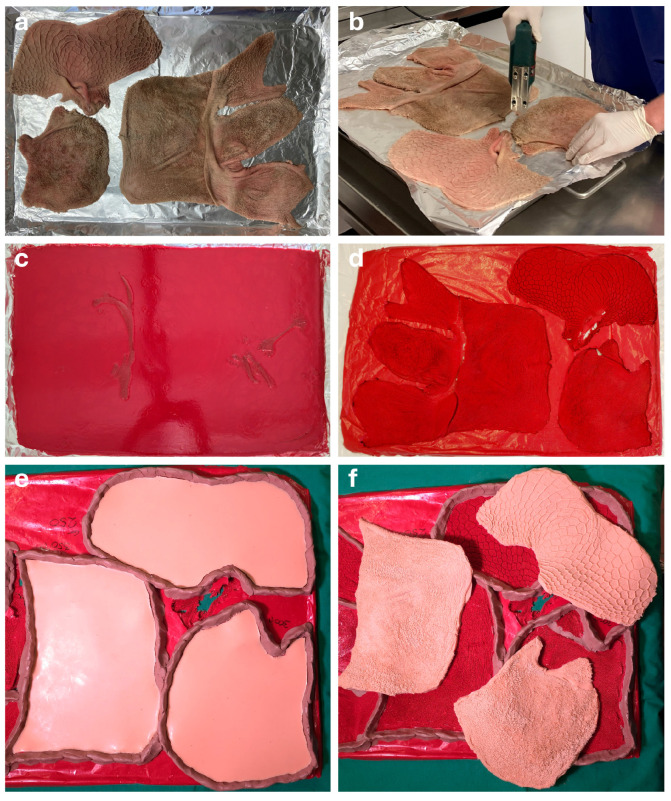
The procedure of surface casting. (**a**) Several pieces of the *Ruminoreticulum* were evened out and placed with the mucosa facing upwards. (**b**) Following, the samples were stapled onto a wooden base beneath aluminum foil to prevent the samples from floating during the casting process. (**c**) Liquid epoxy resin was poured onto the mucosal surface. (**d**) After curing, maceration and drying, the epoxy resin negative molds were placed with the mucosal surface facing upwards. (**e**) Molding clay (purple) was applied to delimit the negative molds, and the preformed areas were filled with liquid silicone (pink). (**f**) After curing, the silicone casts were manually detached from the negative molds.

Since the polymerization of the epoxy resin generates a lot of heat [18], which could damage the organ templates, the applied layer of the epoxy resin needs to be as thin as possible. Therefore, the organ was cut into several pieces and big parts of connective tissue in the *Sulci longitudinales sinister* and *dexter* were removed. This allowed the organ pieces (Figure 1a) to be flattened to a layer thickness of approximately 1 cm, which was optimal for the epoxy resin. In advance of the molding process, the specimens were bathed in 80% ethanol at 20 °C for 5 min [19] to reduce the quantity of the physiologically present microbiome of the *Ruminoreticulum*.

The organ molds were produced in metal trays. To prevent a dislocation of the organ pieces during the application of the epoxy resin, they were stapled to a wooden base with the mucosa facing upwards. A layer of aluminum foil was placed between the organ wall and the wood (Figure 1b) to ensure a better detachment after curing. The epoxy resin mixture was carefully poured onto the mucosal surface (Figure 1c). Polymerization took place at room temperature and lasted between one and two days.

After polymerization, the mold was manually removed from the base. During detachment, large pieces of the organ remained stuck to the wooden base. Following, the epoxy resin mold was macerated by using 32% sodium hydroxide (NaOH) at room temperature [12,20,21] to remove small organ remnants. This took between 6 and 12 h, depending on the specimen. The last remaining organ pieces were removed by using tap water at 40 °C and pulses of strong shower spray [12]. The remaining NaOH was rinsed off using tap water. Finally, the epoxy resin moldings were dried overnight at room temperature (Figure 1d).

#### 2.3.2. Positive Cast

For creation of the positive mucosal cast, the silicone ECOFLEX^®^ 00-50 (KauPo Plankenhorn e.K., Spaichingen, Germany) was used. The silicone was colorized using SILC-PIG^®^ Pantone 488C (KauPo Plankenhorn e.K., Spaichingen, Germany) to resemble the color of light flesh. Before the liquid silicone was applied to the epoxy resin molds, modeling clay was used to limit the flow of the silicone (Figure 1e), and the negative molds were coated with the silicon-based release agent EASE RELEASE™ 205 (KauPo Plankenhorn e.K., Spaichingen, Germany) to support the detachment of the positive casts. Finally, the liquid silicone was applied and allowed to polymerize at room temperature for approximately 5 h.

## 3. Results

### 3.1. Virtual Template and 3D Printing

Virtual templates from three areas of the *Ruminoreticulum* were prepared: *Rumen* (exclusive of the *Atrium*), *Atrium ruminis* and *Reticulum*.

#### 3.1.1. *Rumen* and *Atrium ruminis*

**Figure 2:**
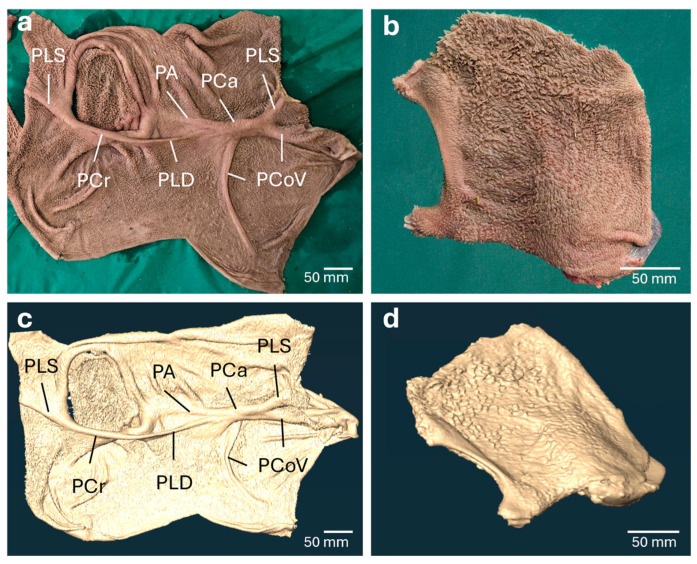
The mucosal surface of the *Rumen* in the anatomical specimen and the corresponding virtual model. The *Rumen* (**a**) and the dorsal organ wall of the *Atrium ruminis* (**b**) were separated from the *Reticulum* and leveled out in advance of computed tomography scanning. In the organ templates, the non-papillated areas of the *Pilae ruminis* (PLS *Pila longitudinalis sinistra*, PCr *Pila cranialis*, PLD *Pila longitudinalis dextra*, PA *Pila accessoria dextra*, PCa *Pila caudalis*, PCoV *Pila coronaria ventralis*) and the areas covered with *Papillae ruminis* can be clearly distinguished. Individual *Papillae* of varying heights and shapes are evident. The papillae-free *Pilae ruminis* can also be identified in the virtual model of the *Rumen* (**c**), thus enabling basic orientation within the model. By their rough appearance, papillated areas are distinguishable from non-papillated areas (**c**,**d**). However, it is obvious that the expression of distinct *Papillae ruminis* is scarce, and the visible *Papillae* are small, broad and cone-shaped.

The papillated areas of the *Rumen* were distinguishable from non-papillated regions, e.g., the *Plica ruminoreticularis,* and the right and left *Pilae longitudinales*. The *Pilae ruminis* and their relative positions to each other were evident, enabling orientation within the virtual template (Figure 2a,c). However, the appearance of characteristic *Papillae ruminis* in the papillated areas (Figure 2a,b) was usually unclear, and the occasionally visible *Papillae ruminis* were small and cone-shaped (Figure 2c,d). Since the virtual templates of the *Rumen* lacked important anatomical features, it was decided not to attempt 3D printing.

#### 3.1.2. *Reticulum*

**Figure 3:**
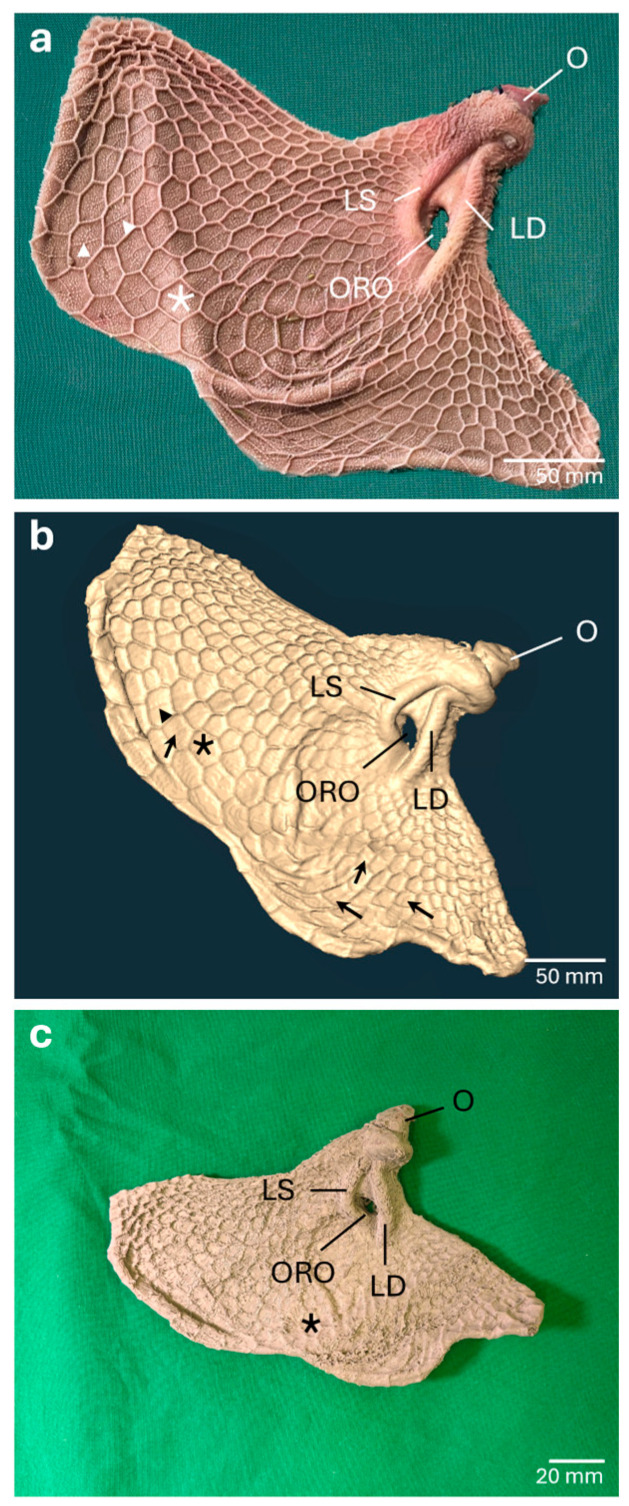
The mucosal surface of the *Reticulum* in the anatomical specimen, the corresponding virtual model and the 1:2 scaled 3D test-print. The isolated *Reticulum* (**a**) was leveled out in advance of computed tomography. The truncated *Oesophagus* (O), the *Sulcus reticuli* delimited by its *Labium sinistrum* (LS) and *Labium dextrum* (LD), the *Ostium reticuloomasicum* (ORO) as well as the *Cellulae reticuli* (asterisk) bordered by *Cristae reticuli* are clearly visible. In addition, small anatomical structures such as the *Papillae reticuli* and small mucosal folds (arrowheads) at the bottom of the *Cellulae* can be identified. In the virtual model (**b**) the *Cristae reticuli* and the formation of the *Cellulae reticuli* can be detected. The small mucosal folds at the bottom of the *Cellulae* (arrowheads) are occasionally visible but can be mistaken with the overall present staircase artifacts (arrows). *Papillae reticuli* cannot be detected. The 3D print (**c**) of the virtual template lacks many anatomical details. Coarser structures like the *Labium dextrum* and *Labium sinistrum sulci reticuli* are expressed, whereas the *Cellulae reticuli* are just barely noticeable. *Papillae reticuli* and the fine mucosal folds cannot be visualized at all.

The virtual template of the *Reticulum* included the truncated *Oesophagus,* the *Sulcus reticuli* and the *Ostium reticuloomasicum*. The *Cristae reticuli* were distinct, allowing the recognition of *Cellulae reticuli*. The *Sulcus reticuli* with its right and left *Labia* as well as the *Ostium reticuloomasicum* were easily identifiable (Figure 3a,b). However, the small *Papillae reticuli*, which regularly occur at the bottom and the walls of the *Cellulae* (Figure 3a), were not observable. The small mucosal folds on the ground of the *Cellulae* (Figure 3a) were sometimes faintly visible but could also be confused with staircasing artifacts (Figure 3b). The template of the *Reticulum* was further processed, but many anatomical details were missing in the 3D print. The *Sulcus reticuli* and the *Ostium reticuloomasicum* were visible, but even *Cristae* and *Cellulae reticuli* were barely recognizable (Figure 3c). The PLA filament used for 3D printing was hard and sturdy, which resulted in an immovable model.

### 3.2. Negative Mold of Mucosal Surface Cast

In total, eight templates were generated. These included the *Saccus dorsalis*, *Saccus ventralis*, right and left *Pilae longitudinales*, *Reticulum*, and *Sulcus reticuli* including the *Cardia* and the *Ostium reticuloomasicum*.

#### 3.2.1. *Rumen*

**Figure 4:**
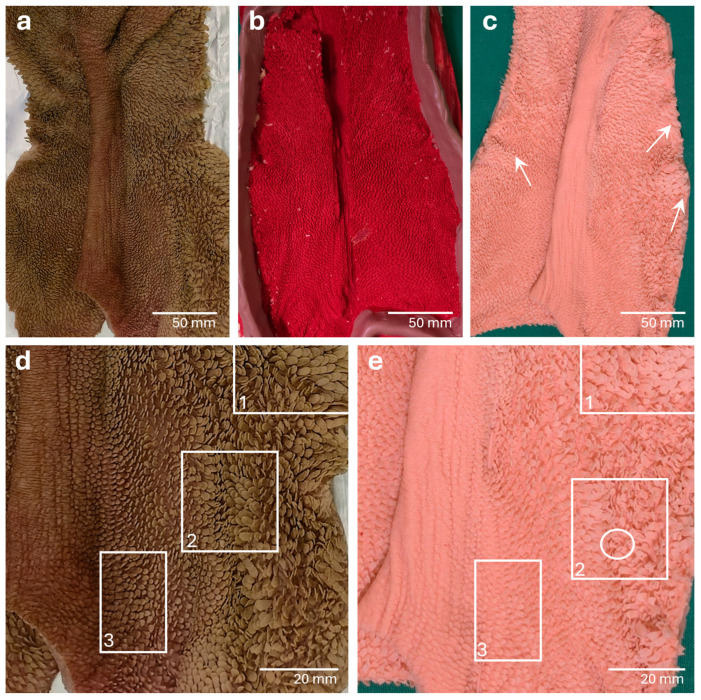
Organ template, negative mold and positive surface cast of the *Rumen.* In the organ template (**a**) a *Pila ruminis* and *Papillae ruminis* with different heights and shapes are visible. The negative mold (**b**) shows a big longitudinal impression of the *Pila ruminis*. Many small holes are visible next to it marking the papillated areas of the specimen. The overview of the resulting positive mucosal cast (**c**) features a *Pila ruminis* and many distinct *Papillae ruminis.* Areas with differently shaped *Papillae* are obvious. The ruminal wall appears wavy in some areas (arrows). In the enlarged pictures of the organ template (**d**) and the positive mucosal cast (**e**), *Papillae ruminis* in different heights and shapes (e.g., leaf-like *Papillae* in square 1, tongue-like *Papillae* in square 2, wart-like *Papillae* in square 3) can clearly be identified. Hollow spaces within the tips of replicated *Papillae* can sometimes be seen (encircled).

In the negative molds of the *Rumen* sections, the *Pilae ruminis* had left prominent impressions. These papillae-free areas could be easily distinguished from the papillae-covered areas, where small holes were visible as impressions of the *Papillae ruminis* (Figure 4b).

#### 3.2.2. *Reticulum*

**Figure 5:**
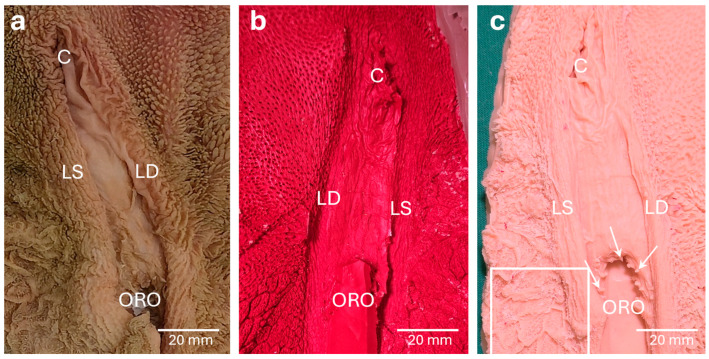
Organ template, negative mold and positive surface cast of the *Sulcus reticuli.* In the organ template (**a**) the *Sulcus reticuli* with its *Labium dextrum* (LD) and *Labium sinistrum* (LS) is visible. This includes also the wrinkled *Cardia* (C) and the *Ostium reticuloomasicum* (ORO). Next to the *Sulcus reticuli*, the reliefs of the ruminal mucosa and the reticular mucosa can be identified on the right side and the left side, respectively. The negative mold (**b**) shows the impressions of all the structures described above. The resulting positive mucosal cast (**c**) reflects all structures of the organ template. Even the *Papillae unguiculiformes* (arrows) can be seen around the *Ostium reticuloomasicum.* In some areas, fine folds of the upper mucosal layer can be seen (square).

**Figure 6:**
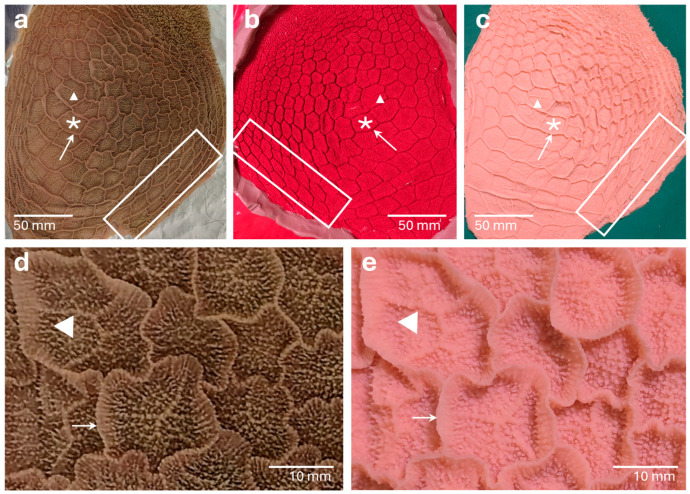
Organ template, negative mold and positive surface cast of the *Reticulum.* In the organ template (**a**), the *Cristae reticuli* (arrow) forming *Cellulae reticuli* (asterisk) are visible. *Papillae reticuli* and small mucosal folds (arrowheads) can be seen on the bottom of the *Cellulae* (**a**,**d**). The negative mold (**b**) shows the impressions of the *Cristae reticuli* as well as the impressions of the *Papillae reticuli* and small mucosal folds on the bottom of the *Cellulae*. The resulting positive mucosal cast (**c**,**e**) displays all structures in the same detail as the original organ. In the overviews (**a**–**c**), distortion of the surface structures is indicated (squares).

In the negative mold of the *Reticulum*, impressions of the *Labium dextrum* and *Labium sinistrum* of the *Sulcus reticuli* were clearly visible, including the *Cardia* and the *Ostium reticuloomasicum.* Next to the *Sulcus reticuli*, the relief of the ruminal and reticular mucosa could be identified (Figure 5b). The formation of the *Cellulae reticuli* was apparent through the impressions of the *Cristae reticuli* (Figure 5b and Figure 6b). Inside the *Cellulae*, the imprints of small mucosal folds and many small holes could be seen as impressions of the *Papillae reticuli* (Figure 6b).

### 3.3. Positive Mucosal Cast

The release agent led to an easy detachment of the silicone casts (Figure 1f), with only little remnants remaining within the impressions. The positive casts resembled the mucosal structure of the organic templates (Figure 4, Figure 5 and Figure 6). Due to the inherent elasticity of the silicone material, the casts remained flexible and deformable, thereby providing a realistic representation of the natural texture and tactile properties of the mucosal surface.

#### 3.3.1. *Rumen*

In the *Rumen*, in addition to the distinct anatomical structures such as the *Pilae ruminis*, the *Papillae ruminis* were remarkably well-differentiated (Figure 4c). Leaf-like, tongue-like and wart-like formed *Papillae* were expressed and could be clearly distinguished from one another. The varying heights of the *Papillae* in the different areas of the *Rumen* could also be easily seen (Figure 4e). In some areas, the flat ruminal wall appeared wavy (Figure 4c), and in other areas, fine folds were visible in the upper mucosal layer. Additionally, small hollow spaces occurred within some *Papillae* (Figure 4e).

#### 3.3.2. *Reticulum*

In addition to larger anatomical structures such as the *Sulcus reticuli* with its right and left *Labia,* the wrinkled *Cardia* (Figure 5c) and the *Cristae reticuli* (Figure 6c,e), finer anatomical structures were also apparent. *Papillae reticuli* were visible on the walls and ground of the *Cellulae reticuli* and the small mucosal folds on the floor of the *Cellulae* were noticeable (Figure 6c,e). Even the *Papillae unguiculiformes* at the margin of the *Ostium reticuloomasicum,* were recognizable (Figure 5c). In addition, regional distortions of the mucosal surface occurred (Figure 6c), and fine folds appeared in some areas of the upper mucosal layer (Figure 5c).

## 4. Discussion

In the current study, we successfully produced high-fidelity replicas of the mucosal surfaces of the goat’s *Rumen* and *Reticulum*. One limitation of the resulting model is that it reflects only the anatomical features of a single animal of a specific breed and sex. In principle, a model can always represent only one of many possibilities. It is therefore important to verify whether the characteristics of the selected animal are typical of the species. Following examination and dissection, we concluded that the anatomy of the animal’s *Ruminoreticulum* was typical of a goat.

The results of the present study demonstrated clear advantages of the surface casting method compared to 3D printing.

### 4.1. Virtual Template and 3D Printing

The CT-based virtual templates showed that fine anatomical structures of the mucosal surfaces like the *Papillae* in *Rumen* and *Reticulum* could not be displayed in detail. In the *Rumen*, the bases of the *Papillae* touched each other, resulting in a virtual fusion over most of their length. Only the tips of some *Papillae* were visible, giving the impression of an uneven surface with only a few distinct *Papillae*. This was caused by the partial volume effect, which led to a shortening of the *Papillae* lengths and a widening of the papillary bases [22,23].

It is debatable whether magnet resonance imaging could result in a better visualization of the *Papillae* in the *Rumen* and the *Reticulum*. Theoretically, the enhanced soft tissue contrast [24] could enable the differentiation between single *Papillae*. However, due to the lower spatial resolution [24], the partial volume effect intensifies [23] and inhibits the depiction of detailed *Papillae*. Additionally, the lower spatial resolution leads to stronger staircasing artifacts due to an increased distance of the individual slices, which prevents the development of high-fidelity 3D templates.

Another approach of generating virtual templates is the usage of 3D surface scanners. This technique depicts the information of the outer surface with high accuracy and thus enriches virtual templates in superficial form and most notably in accurate colorization of the scanned specimens [25]. This method could eventually provide an interesting alternative to the generation of templates of the *Reticulum.* However, overlapping structures, like the *Papillae ruminis*, cannot be displayed separately from another, which assumably results in an inferior haptic sensation, compared to the mucosal surface casting technique.

The 3D print of the *Reticulum* was neither visually nor haptically convincing. To imitate the haptic characteristics of organs, elastic filaments for 3D printing are available, which are superior to sturdy filaments concerning tactile experiences of soft tissue specimen [26,27,28]. Nevertheless, the low resolution of the virtual templates prevents adequate visualization of small anatomical structures. This led to a poorly detailed 3D print, because the resulting output cannot overcome the deficiencies of the virtual templates [27].

### 4.2. Surface Casting

Using the surface casting method, high-fidelity replicas of the mucosal surface of the *Rumen* and *Reticulum* were obtained.

The use of epoxy resin has proven to be suitable for producing negative molds. The low viscosity of the BIODUR^®^ E20 PLUS favored an upright positioning of the *Papillae ruminis* and enabled easy flow between the individual *Papillae*. This allowed the *Papillae* to leave separate impressions in the negative mold, which was required for the display of individual *Papillae* in the positive cast. Their different heights and shapes were also noticeable. Furthermore, the slightly flexible character of molds created with BIODUR^®^ E20 PLUS prevented damaging of the templates during the whole process. Additionally, the resin is resistant to corrosive chemicals. One disadvantage, however, is the significant heat generation during polymerization. Accordingly, complete casting of the *Ruminoreticulum* was not feasible, precluding full representation of both the mucosal surface architecture and overall organ morphology. The organ was therefore dissected, and representative sections were flattened prior to casting. This approach minimized epoxy resin layer thickness, thereby reducing polymerization-associated heat generation and preserving tissue integrity. The flat layout of the rather rounded organs has proven to be essential for the generation of mucosal surface casts, albeit it causes distorted representations of the mucosa in some areas. To preserve the integrity of the organ templates during the polymerization process, they were washed with 80% ethanol. This caused a tonic contraction of the smooth muscle cells in the *Lamina muscularis mucosae* [29,30], which manifested regionally as fine folds in the upper mucosal layer. Omitting alcohol could prevent this artifact, but it remains unclear whether the organ tissue would remain intact during the curing time of the epoxy resin otherwise. Additionally, trapped air in the impressions of the negative mold formed bubbly artifacts in some *Papillae ruminis* of the positive mucosal cast. These artifacts must be considered when visually examining the positive mucosal casts, yet they do not affect the haptic properties.

The silicone used for the positive cast was suitable for penetrating even the smallest impressions in the epoxy resin templates. This enabled the development of life-like casts of the mucosal surface of the *Rumen* and *Reticulum*. As described above, in addition to the individual *Papillae* in the *Rumen* and *Reticulum*, the different shapes and heights of the *Papillae ruminis* could also be depicted. Owing to their flexibility, the silicone casts exhibit tactile properties that closely resemble those of the native organ.

In summary, the generation of virtual templates cannot yet meet the standards set by the mucosal surface casting technique. However, the manufacturing technologies, like 3D-printers and 3D-printing material, and the imaging techniques are constantly and rapidly evolving and can be potentially reconsidered as an alternative in the future.

## 5. Conclusions

In conclusion, the creation of a virtual template based on CT images was not suitable to produce a 3D printed model of the mucosal surface of the *Rumen* and the *Reticulum* in the desired detail. In contrast, the surface casting method enabled the production of detailed and lifelike replicas of the mucosal surfaces. Although there were a few minor artifacts visible, the models have no limitations in terms of haptic impression and are visually very close to the native organ. This makes the surface casts ideal for the interior lining of the planned 3D forestomach simulator. This simulator is designed to allow students to practice palpating the *Rumen* and *Reticulum* before working on living animals, which improves the welfare of the animals used in this exercise. If, for legal reasons, this exercise is no longer possible on live animals, the simulator offers a good alternative to living rumen-fistulated ruminants.

Furthermore, this technique is generally suitable to create highly detailed, haptically convincing models of the inner and outer surfaces of soft tissue specimens. These models can be used in veterinary education as a supplement to live animals and animal cadavers, thereby helping to reduce the number of animals used.

## Data Availability

The original contributions presented in this study are included in the article. Further inquiries can be directed to the corresponding author.

## Abbreviations

The following abbreviations are used in this manuscript:

3D	Three-dimensional
CAD	Computer-Aided Design
CT	Computed tomography
STL	Stereolithography
MEK	Methylethylketone
NaOH	Sodium hydroxide
PLS	*Pila longitudinalis sinistra*
PCr	*Pila cranialis*
PLD	*Pila longitudinalis dextra*
PA	*Pila accessoria dextra*
PCa	*Pila caudalis*
PCoV	*Pila coronaria ventralis*
O	*Oesophagus*
LS	*Labium sinistrum*
LD	*Labium dextrum*
ORO	*Ostium reticuloomasicum*
C	*Cardia*
